# Development and validation of the 3D U-Net algorithm for segmentation of pelvic lymph nodes on diffusion-weighted images

**DOI:** 10.1186/s12880-021-00703-3

**Published:** 2021-11-13

**Authors:** Xiang Liu, Zhaonan Sun, Chao Han, Yingpu Cui, Jiahao Huang, Xiangpeng Wang, Xiaodong Zhang, Xiaoying Wang

**Affiliations:** 1 Department of Radiology, Peking University First Hospital, No.8 Xishiku Street, Xicheng District, Beijing, 100034 China; 2 Beijing Smart Tree Medical Technology Co. Ltd., No.24, Huangsi Street, Xicheng District, Beijing, 100011 China

**Keywords:** Prostate cancer, Lymph nodes, Segmentation, Detection, Diffusion-weighted imaging, Deep learning, U-Net

## Abstract

**Background:**

The 3D U-Net model has been proved to perform well in the automatic organ segmentation. The aim of this study is to evaluate the feasibility of the 3D U-Net algorithm for the automated detection and segmentation of lymph nodes (LNs) on pelvic diffusion-weighted imaging (DWI) images.

**Methods:**

A total of 393 DWI images of patients suspected of having prostate cancer (PCa) between January 2019 and December 2020 were collected for model development. Seventy-seven DWI images from another group of PCa patients imaged between January 2021 and April 2021 were collected for temporal validation. Segmentation performance was assessed using the Dice score, positive predictive value (PPV), true positive rate (TPR), and volumetric similarity (VS), Hausdorff distance (HD), the Average distance (AVD), and the Mahalanobis distance (MHD) with manual annotation of pelvic LNs as the reference. The accuracy with which the suspicious metastatic LNs (short diameter > 0.8 cm) were detected was evaluated using the area under the curve (AUC) at the patient level, and the precision, recall, and F1-score were determined at the lesion level. The consistency of LN staging on an hold-out test dataset between the model and radiologist was assessed using Cohen’s kappa coefficient.

**Results:**

In the testing set used for model development, the Dice score, TPR, PPV, VS, HD, AVD and MHD values for the segmentation of suspicious LNs were 0.85, 0.82, 0.80, 0.86, 2.02 (mm), 2.01 (mm), and 1.54 (mm) respectively. The precision, recall, and F1-score for the detection of suspicious LNs were 0.97, 0.98 and 0.97, respectively. In the temporal validation dataset, the AUC of the model for identifying PCa patients with suspicious LNs was 0.963 (95% CI: 0.892–0.993). High consistency of LN staging (Kappa = 0.922) was achieved between the model and expert radiologist.

**Conclusion:**

The 3D U-Net algorithm can accurately detect and segment pelvic LNs based on DWI images.

## Background

More than 15% of prostate cancer (PCa) patients were confirmed to have lymph node (LN) invasion during radical prostatectomy [1]. Patients with regional pelvic LN metastases face an increasing risk of mortality and should be treated aggressively [2]. Therefore, the detection of metastatic LNs is crucial for appropriate treatment selection and management. Precise LN staging allows urologists to determine which patients may benefit from a pelvic LN dissection (PLND) during radical prostatectomy and which patients may safely avoid it [3–5].

Multiparametric MRI (mpMRI) has been reported to play a central role in detecting and staging PCa [6, 7]. Diffusion-weighted imaging (DWI) is characterized by a high contrast between the metastatic lesion and healthy tissue, thus yielding excellent efficiency in primary tumour evaluation and LN identification [8]. Radiologists usually regard LNs with a short diameter above 0.8 cm on DWI images as suspicious for metastatic LNs, and this should be highlighted in the radiology report [9, 10]. However, the detection of metastatic LNs on DWI images by radiologists is time-consuming and demands experience.

Recently, convolutional neural networks (CNNs) have emerged as a revolution in the field of image analysis. CNN is based on the concept that deep learning-based methods can provide an automated diagnosis and quantitative assessment with high reproducibility, thus allowing more objective reporting [11, 12].

Cuocolo et al. [13] compared different deep learning methods (U-Net, efficient neural network (ENet), and efficient residual factorized ConvNet (ERFNet)) for whole-gland and zonal prostate segmentation on T2WI images. Their results showed that ENet achieved the best Dice similarity score and U-Net for the second place. Comelli et al. [14] compared the performance of the U-Net and E-Net for lung segmentation on high-resolution computerized tomography images. The dice similarity coefficient showed no statistical differences between segmentation methods (95.9% vs. 95.61%, *P* = 0.68). Multiple studies have been published on the development of U-Net-based LN segmentation methods, most of which use CT [15, 16], PET/CT [17, 18], or MR lymphography images [19] rather than DWI images.

In this study, we introduced the 3D U-Net framework for automatically detecting and segmenting suspicious pelvic LNs in DWI images and then evaluated the framework on an hold-out test dataset. We hope that the automated detection and segmentation of LNs will lay a foundation for the comprehensive automated analysis of tumour burden in PCa patients.

## Materials and methods

This retrospective study was performed with permission from the local institutional ethics committee. The need for written informed consent was waived.

### Study subjects

A dataset of 425 patients suspected of having PCa between January 2019 and December 2020 was acquired from the local picture archiving and communication system (PACS) for algorithm development. The inclusion criteria were as follows: (1) patients with clinically suspected PCa (elevated PSA); (2) patients without any prior treatment of PCa (such as androgen deprivation, radical prostatectomy, or radiation therapy); and (3) patients whose high b-value (1000 or 800 s/mm^2^) DWI images were available. Fifteen patients were excluded due to unqualified MRI quality, 6 patients were excluded for obvious and massive metastatic involvement (e.g., obvious destruction of pelvic or bony structures), and 11 patients with a history of pelvic surgery were not included in the analysis. A total of 393 patients were finally recruited for 3D U-Net algorithm development, including 56 patients with PI-RADS scores of 1–2, 14 patients with PI-RADS scores of 3, and 323 patients with PI-RADS scores of 4–5.

Another dataset of 77 patients with clinically suspected PCa seen between January 2021 and April 2021 was enrolled to externally evaluate the proposed algorithm. Among them, 37 patients had at least one suspicious metastatic LN (above 0.8 cm in the short-axis dimension and high signal intensity on DWI), and 40 patients did not have visible suspicious LNs. All mpMRI data were deidentified before inclusion, and the clinical information, such as age and prostate-specific antigen (PSA) level, of each enrolled patient was recorded.

### Pelvic mpMRI

The mpMRI examinations were performed with three 3.0-Tesla scanners (Achieva, Philips Healthcare; Discovery, GE Healthcare; Interia, Philips Healthcare) using a phased-array coil. The standard mpMRI protocol at our institution included a combination of T2WI, T1WI, DWI, and dynamic contrast-enhanced imaging. Details of the imaging parameters of the DWI sequence are summarized in Table 1.

**Table 1:** Imaging protocols of the pelvis DWI sequences

Protocols	3.0 T Achieva (Philips Healthcare, the Netherlands)	3.0 T Discovery (Ge healthcare, Milwaukee, WI)	3.0 T Interia (Philips Healthcare, the Netherlands)
B-values (s/mm^2^)	0, 800	0, 800	0, 1000
TR/TE (ms)	3400/54	3000/60	4959/78
Imaging matrix	224 × 224	256 × 256	240 × 240
Field of view (mm^2^)	375 × 375	360 × 400	360 × 400
Slice thickness (mm)	6	8	7
Number of slices	24	25	28
Intersection gap	No	No	No

*TR*  Repetition time, *TE* Echo time

### Manual annotations

Before annotation, all the Digital Imaging and Communication in Medicine (DICOM) format images were converted to NIFTI format. Images were manually annotated with ITK-SNAP (version 3.6; Penn Image Computing and Science Laboratory, Philadelphia, PA).

In the algorithm development dataset, all discernible LNs on DWI images were manually annotated by two junior radiologists (both with 4 years of reading experience) within the pelvic region (namely, Mask 1 and Mask 2). An expert radiologist (with more than 15 years of reading experience) subsequently modified the two sets of manual annotations (Mask 3 and Mask 4, respectively). The Dice score was used to evaluate the reliability of the manual annotations between different masks.

### Ground truth

**Fig. 1:**
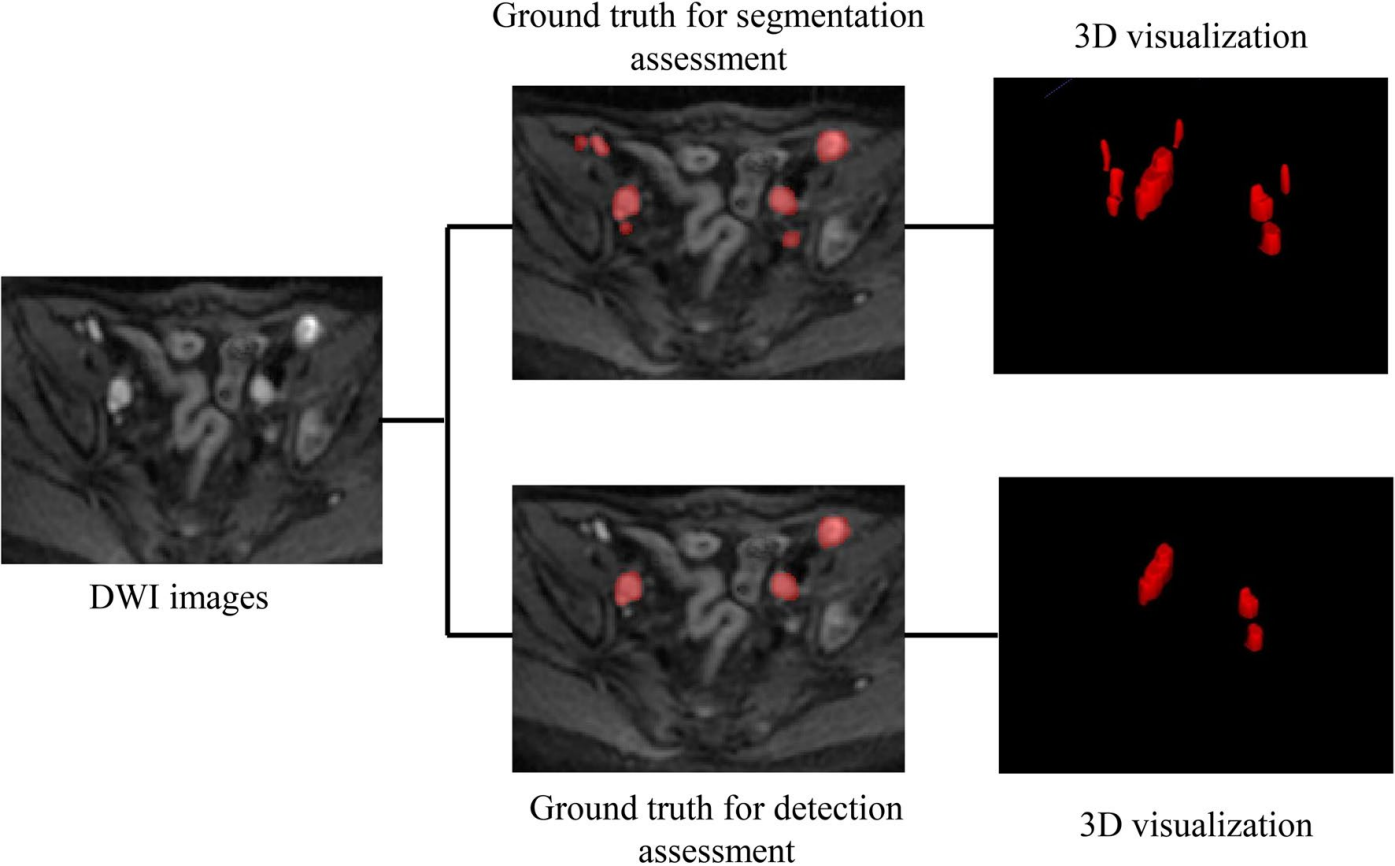
Ground truth for model evaluation. The first row shows the ground truth for segmentation of the model, and the second row shows the ground truth for detection assessment of the model

For model development, the manual annotations of the pelvic LNs were regarded as ground truth for the assessment of segmentation (Mask 4). LNs with short diameters > 0.8 cm were regarded as suspicious metastatic LNs, and their annotations were taken as ground truth for the assessment of LN detection (Fig. 1). For temporal validation, the manual annotations of the suspicious metastatic LNs, edited by one of the junior radiologists under the supervision of the expert radiologist, were regarded as ground truth for the segmentation and detection assessment. The LN staging performed by the expert radiologist was considered ground truth for the consistency evaluation of the N-staging between the model and the radiologist.

### Model development

The CNN developed for the segmentation of pelvic LNs on DWI images is the 3D U-Net [20], which replaced all 2D operations (convolution kernels, pooling layers, and upconvolution kernels) of the U-Net architecture with 3D counterparts. By taking full advantage of the 3D spatial information, the algorithm can learn typical features with higher discrimination capability than those learned from 2D CNNs.

**Fig. 2:**
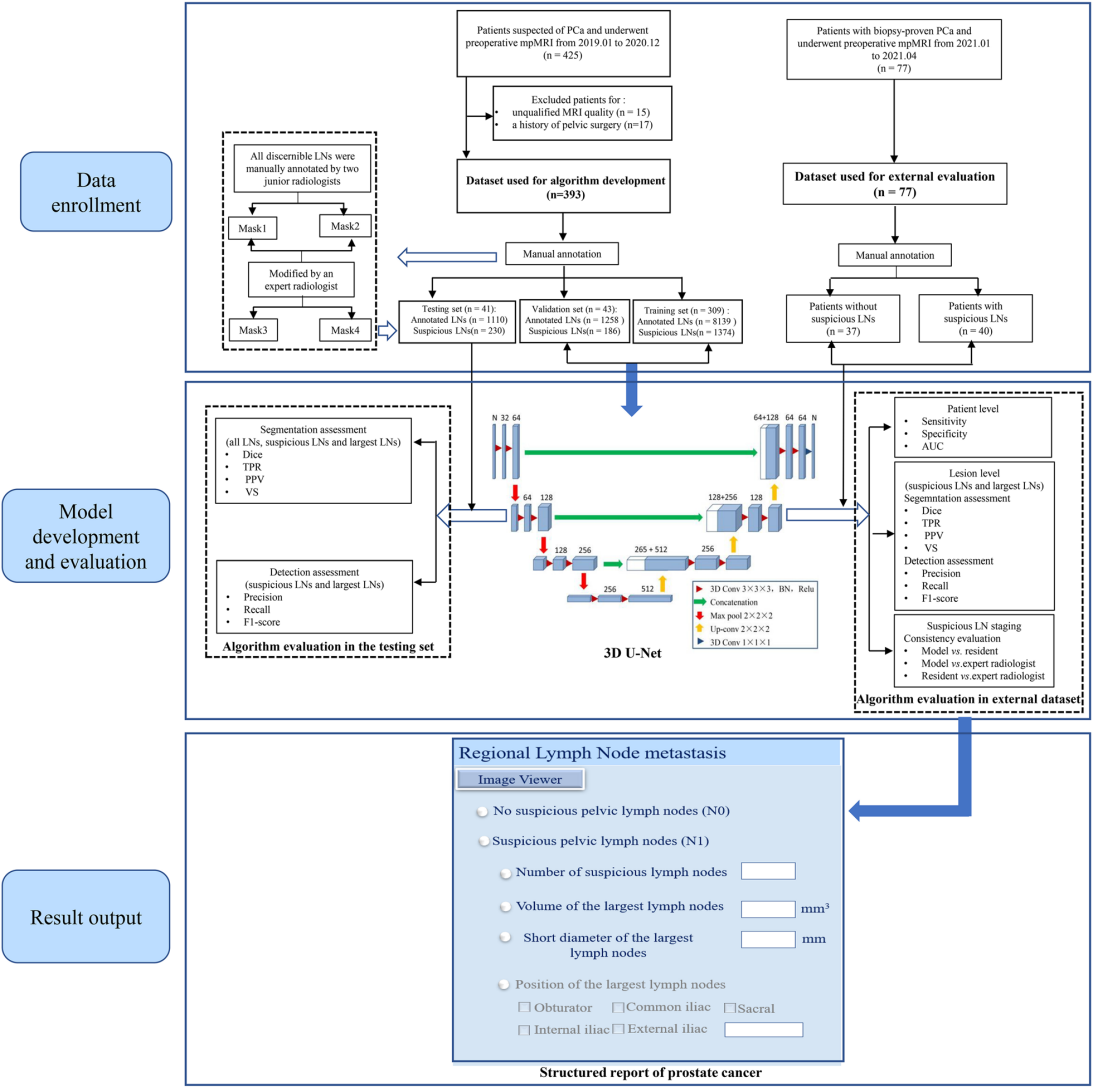
The flowchart of the algorithm development and result output

The 3D U-Net was trained with DWI images and their corresponding manual annotations. The 393 patients were randomly divided into the training (n = 309), validation (n = 43) or testing (n = 41) set at a ratio of 8:1:1. The hold-out test dataset (n = 77) was used to further evaluate the detection and segmentation accuracy of the 3D U-Net model (Fig. 2).

All input images were resized to 64 × 256 × 256 (z, y, x) before training to maintain the optimal image features. To train the 3D U-Net segmentation models, we exploited the ADAM optimizer with an initial learning rate of 10^−4^ and a fixed batch size of 10 images. Skewing (angel: 0–5), shearing (angel: 0–5) and translation (scale: − 0.1, 0.1) of the images were applied for data augmentation. U-Net was trained for 300 epochs until the validation loss function failed to rise. The network was written in Python (version 3.6). The PyTorch (version 0.4.1) deep learning platform was employed for training and validation. All experiments were performed using an NVIDIA Tesla P100 16G, running on Ubuntu.

### Quantitative measurement of LNs and output to the radiology report

A cluster of contiguous voxels predicted by the algorithm was designated as an individual LN. After receiving the segmentation result of the CNN, the volume and short diameter of each segmented LN were automatically calculated via the minimum-volume bounding box method. The number of suspicious LNs (short diameter > 0.8 cm), as well as the volume and short diameter of the largest LN, were automatically sent to the structured report (Fig. 2). If at least one suspicious LN was detected by the model, then the N-staging was automatically inputted as N1 to the radiology report. In contrast, if no suspicious LN was detected, the N-staging was automatically inputted as N0 to the radiology report.

### Model performance evaluation

Two datasets were collected in this study—the algorithm development dataset with manual annotations for all LNs and the hold-out test dataset with manual annotations only for suspicious LNs. Consequently, two sets of evaluation metrics were formulated to evaluate the algorithm.

### Evaluation using the model development dataset

The ability of the 3D U-Net algorithm to segment and detect individual LNs was evaluated by comparison of the automated annotation with manual annotation. According to the overlap between manual annotation and automated segmentation, the voxels of the image can be defined as true positives (TPs), false positives (FPs), true negatives (TNs), and false negatives (FNs). The segmentation performance of the model was assessed using Dice, positive predictive value (PPV), true positive rate (TPR), volumetric similarity (VS), Hausdorff distance (HD), the Average distance (AVD), and the Mahalanobis distance (MHD) [21], which were defined by:

$$\begin{aligned} Dice &=\frac{2TP}{2TP+FP+FN}\\ PPV &=\frac{TP}{TP+FP}\\TPR &=\frac{TP}{TP+FN}\\VS&=1-\frac{|FN-FP|}{2TP+FP+FN}\\HD(A, B)& = max(h(A, B), h(B,A))\\ AVD(A, B) &= max(d(A, B), d(B,A)) \\MHD(X, Y) &=\sqrt{(\mu x - \mu y)TS-1(\mu x - \mu y)}\end{aligned}$$

where h(A, B) is called the directed Hausdorff distance between two finite point sets A and B, d(A, B) is the directed Average Hausdorff distance between A and B, μx and μy are the means of the point sets.

The average short diameter and volume of LNs in manual segmentation and automated segmentation were calculated to further quantitatively estimate the segmentation efficacy of the 3D U-Net algorithm.

Based on the segmentation results, we proposed a detection approach for suspicious LNs. A suspicious LN was considered to be correctly detected when overlap existed between automated segmentation and manual annotation. The precision, recall and F1-score (harmonic mean of precision and recall) were then calculated to evaluate the detection accuracy of the model. The precision and recall values reflected the percentage of correctly segmented LNs out of all the identified LNs [TP/(TP + FP)] and the percentage of correctly segmented LNs out of all the annotated LNs [TP/(TP + FN)], respectively. The segmentation performance (all LNs, suspicious LNs, and largest LNs) and detection performance (suspicious LNs and largest LNs) of the 3D U-Net were assessed in the testing set.

### Evaluation using the hold-out test dataset

The hold-out test dataset was used to evaluate the ability of the model to discriminate between 37 patients with suspicious LNs and 40 patients without suspicious LNs. Sensitivity, specificity, and area under the receiver operating characteristic curve (AUC) were used to assess the performance of the algorithm at the patient level. Then, the segmentation performance (Dice, PPV, and TPR) and detection performance (precision, recall, and F1-score) of the model of suspicious LNs and largest LNs were assessed at the lesion level.

To further evaluate the clinical application value of the model of suspicious LN evaluation, the LN staging (N0 or N1) of the model was compared with the interpretation of the radiologists (one resident with less than 2 years of reading experience and one expert radiologist with more than 15 years of reading experience).

### Statistical analysis

MedCalc (version 14.8; MedCalc Software, Ostend, Belgium) and SPSS (version 22.0, IBM Corp., Armonk, NY, USA) were used for the statistical analyses. Numerical data were averaged over all patients and reported as the mean ± standard deviation (SD). One-way analysis of variance (ANOVA) was used to compare patient characteristics (age, PSA level, LN volume, and short diameter) and the segmentation performance of the algorithm (Dice, TPR, PPV, VS, HD, AVD and MHD) among different groups, and the least significant difference (LSD) was used for post hoc multiple comparisons. Paired *t*-tests, Pearson correlation, and Bland–Altman analyses were performed to compare the manual versus automated determination of the short diameter and volume of the LNs. Cohen’s kappa coefficient was used to assess the consistency of LN staging between the model and the radiologists in the hold-out test dataset. *P* < 0.05 was considered indicative of a statistically significant difference.

## Results

### Patient and LN characteristics

The patients’ characteristics are presented in Table 2. In the model development dataset, a total of 10,507 visible LNs were annotated: 8139 in the training set (27 per patient on average), 1258 in the validation set (29 per patient on average), and 1110 in the testing set (27 per patient on average). A total of 201 suspicious metastatic LNs were annotated in the hold-out test dataset of 37 PCa patients.

**Table 2:** The characteristics of patients and lymph nodes

Characteristics	Model development dataset				Hold-out test dataset			*P* value
	Training set	Validation set	Testing set	*P* value	Patients with suspicious LNs	Patients without suspicious LNs	*P* value	
No. of patients	309	43	41	–	37	40	–	–
Age, mean ± SD (years)	70.3 ± 9.6	71.2 ± 7.3	70.8 ± 8.8	0.785	69.4 ± 8.8	71.4 ± 8.4	0.305	0.990
PSA, median (ng/ml)	13.00 (7.13,23.43)	14.21 (8.20, 26.73)	12.20 (7.42, 21.30)	0.552	15.49 (9.25, 26.65)	10.69 (7.10, 18.82)	0.031	0.088
No. of annotated LNs	8139	1258	1110	–	–	–	–	–
Average LNs per patient	27 (5, 30)	29 (6, 33)	27 (3, 35)	0.537	–	–	–	–
No. of suspicious LNs	1374	186	230	–	201	–		
Short diameter of largest LNs (cm)	0.83 ± 0.30	0.93 ± 0.33	0.83 ± 0.29	0.145	–	–	–	–
Volume of largest LNs (cm^3^)	9.03 (4.60, 17.32)	10.93 (5.30, 17.40)	7.09 (5.37, 14.08)	0.628	–	–	–	–
Scanners								
3.0 T Achieva	96	16	12	–	12	15	–	–
3.0 T Discovery	113	13	15	–	14	13	–	–
3.0 T Interia	100	14	14	–	11	12	–	–

*PSA* prostate-specific antigen, *LN* lymph node

There was no significant difference in age or PSA level between the model development dataset and the hold-out test dataset (*P* = 0.990 and 0.088, respectively). The PSA level between patients with or without suspicious LNs showed a significant difference (*P* = 0.031) in the hold-out test dataset. There was no significant difference in the number of annotated LNs, short diameter of the largest LNs, or volume of the largest LNs among the three sets in the model development dataset (*P* = 0.537, 0.145, and 0.628, respectively).

### Reliability of the manual annotations

The inter- and intrareader reliabilities of the manual annotations were assessed based on the Dice score. The Dice scores between different masks are as follows: Mask 1 *vs.* Mask 2: 0.75 ± 0.03; Mask 1 *vs.* Mask 3: 0.78 ± 0.05; Mask 2 *vs.* Mask 4: 0.80 ± 0.09; and Mask 3 *vs.* Mask 4: 0.88 ± 0.06. The high Dice scores between Mask 3 and Mask 4 confirmed the reliability of the manual annotations.

### Segmentation performance of the model

**Fig. 3:**
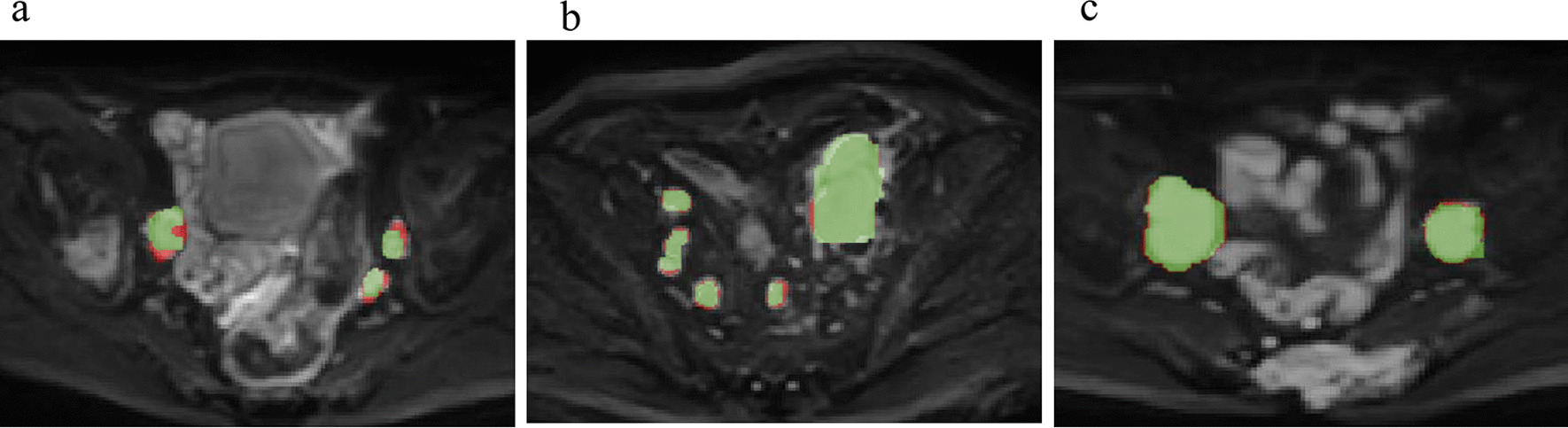
Examples of the segmentation results of the 3D U-Net for the lymph nodes. **a** The automated segmentation of LNs with a Dice score of 0.78; **b** The automated segmentation of LNs with a Dice score of 0.85; **c** The automated segmentation of LNs with a Dice score of 0.93 (red label: manual annotation; green label: automated segmentation)

The LN segmentation accuracy was evaluated in the testing set. As shown in Table 3, the model achieved optimal segmentation performance for the largest LNs with the highest Dice, TPR, PPV, VS, HD, AVD and MHD values of 0.88 ± 0.15, 0.89 ± 0.21, 0.83 ± 0.16, 0.88 ± 0.20, 2.02 ± 0.09 (mm), 2.01 ± 0.07 (mm), 1.54 ± 0.12 (mm) respectively. The metrics of the segmentation accuracy of all LNs were significantly lower than those of the suspicious LNs and the largest LNs (all with *P* < 0.05). There was no significant difference in the metrics between the suspicious and largest LNs (all with *P* > 0.05). There was no significant difference among different scanners concerning the segmentation accuracy of all LNs (all with *P* > 0.05). Exemplary cases of segmentation of LNs with different Dice scores are shown in Fig. 3.

**Table 3:** Segmentation accuracy of the 3D U-Net algorithm in the testing set (n = 41)

Metrics	All LNs in different scanners				All LNs	Suspicious LNs	Largest LNs	*P* value		
	3.0 T Achieva	3.0 T Discovery	3.0 T Interia	*P* value						
								All vs Suspicious	All vs Largest	Suspicious vs Largest
Dice (95% CI)	0.79 ± 0.08 (0.73, 0.85)	0.75 ± 0.17 (0.67,0.81)	0.75 ± 0.17 (0.67, 0.83)	0.779	0.76 ± 0.15 (0.71, 0.81)	0.85 ± 0.13 (0.79, 0.91)	0.88 ± 0.15 (0.81, 0.94)	0.009	0.002	0.496
TPR (95% CI)	0.77 ± 0.13 (0.68, 0.86)	0.73 ± 0.20 (0.66, 0.80)	0.77 ± 0.20 (0.64, 0.90)	0.813	0.76 ± 0.18 (0.69, 0.83)	0.82 ± 0.26 (0.78, 0.86)	0.89 ± 0.21 (0.80, 0.98)	0.017	0.012	0.045
PPV (95% CI)	0.72 ± 0.09 (0.67,0.77)	0.74 ± 0.18 (0.69, 0.79)	0.75 ± 0.21 (0.66, 0.84)	0.712	0.73 ± 0.17 (0.66, 0.80)	0.80 ± 0.20 (0.72, 0.88)	0.83 ± 0.16 (0.76, 0.90)	0.014	0.005	0.394
VS (95% CI)	0.81 ± 0.09 (0.77, 0.85)	0.83 ± 0.15 (0.80, 0.86)	0.84 ± 0.16 (0.78, 0.90)	0.453	0.82 ± 0.14 (0.78, 0.86)	0.86 ± 0.19 (0.79, 0.93)	0.88 ± 0.20 (0.81, 0.95)	0.046	0.013	0.576
HD(mm) (95% CI)	3.46 ± 0.22 (3.31, 3.61)	3.27 ± 0.18 (2.38, 4.16)	3.51 ± 0.45 (3.21, 3.81)	0.213	3.41 ± 0.67 (2.97, 3.85)	2.56 ± 0.18 (1.69, 3.43)	2.02 ± 0.09 (1.30, 2.74)	0.039	0.021	0.521
AVD(mm) (95% CI)	3.03 ± 0.07 (2.44, 3.62)	2.98 ± 0.05 (2.31, 3.65)	3.26 ± 0.15 (2.44, 4.08)	0.389	3.09 ± 0.17 (2.40, 3.78)	2.03 ± 0.27 (1.24, 2.82)	2.01 ± 0.07 (1.60,2.42)	0.027	0.031	0.865
MHD(mm) (95% CI)	2.34 ± 0.14 (2.15, 2.53)	2.51 ± 0.08 (2.26, 2.76)	2.67 ± 0.16 (2.24, 3.10)	0.132	2.51 ± 0.35 (1.67, 3.35)	1.62 ± 0.15 (1.27, 1.97)	1.54 ± 0.12 (1.25, 3.37)	0.035	0.028	0.670

Suspicious LNs indicates the LNs larger than 0.8 cm in the shortest diameter

*LN* lymph node, *PPV* positive predictive value, *TPR* true positive rate, *VS* volumetric similarity, *HD* Hausdorff distance, *AVD* Average distance, *MHD* Mahalanobis distance

### Quantitative evaluation of segmentation performance

**Fig. 4:**
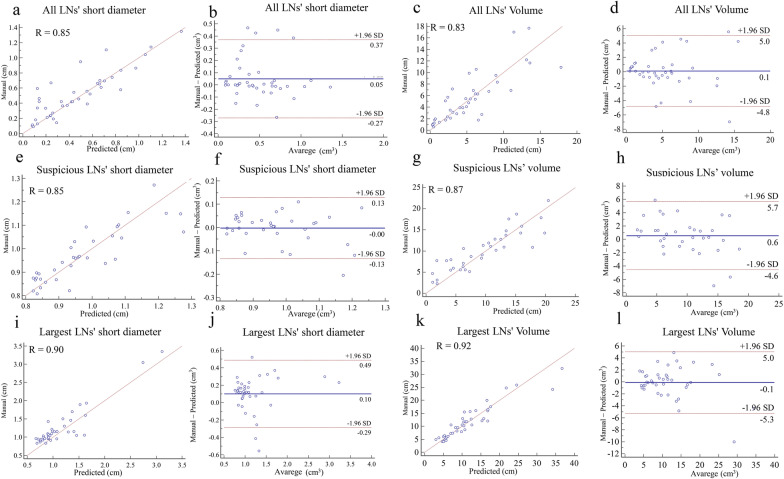
Quantitative comparisons of the LNs' short diameter and volume. Correlation and Bland–Altman plots of LNs' short diameter and volume between automated segmentation and manual segmentation for all LNs (**a**–**d**), suspicious LNs (**e**–**h**), and largest LNs (**i**–**l**)

The average short diameter and volume measurements for all LNs, suspicious LNs, and largest LNs are summarized in Table 4. Quantitative comparisons between automated and manual segmentation are shown in Fig. 4. Both the short diameter and volume of the automatically segmented LNs showed a close correlation (all with R > 0.80) with manually annotated LNs. The Bland–Altman analysis of short diameter and volume showed good consistency between the automated segmentation and manual annotation of all LNs, suspicious LNs, and largest LNs, and most values were within the consistency interval.

**Table 4:** Quantitative measurements between automated segmentation and manual annotation in the testing set (n = 41)

Quantitative metrics	All LNs			Suspicious LNs			Largest LNs		
	Automated segmentation	Manual annotation	*P* value	Automated segmentation	Manual annotation	*P* value	Automated segmentation	Manual annotation	*P* value
Volume (cm^3^)	5.35 ± 3.71	5.45 ± 2.79	0.829	9.54 ± 3.88	10.09 ± 2.66	0.709	11.78 ± 5.25	11.67 ± 6.04	0.887
Short diameter (cm)	0.46 ± 0.30	0.51 ± 0.31	0.482	0.95 ± 0.29	0.99 ± 0.20	0.492	1.12 ± 0.50	1.22 ± 0.53	0.810

*LN* lymph node

### LN detection based on segmentation

Detection of suspicious LNs and the largest LNs in the testing set are shown in Table 5. The developed method achieved good performance in the detection of suspicious LNs with a precision of 0.97 (226/231), recall of 0.98 (226/230), and F1-score of 0.97. Moreover, all 41 of the largest LNs from 41 PCa patients were correctly detected by the algorithm.

**Table 5:** Detection accuracy of lymph nodes in the testing set (n = 41)

	Suspicious LNs	Largest LNs
True positive	226	41
False Positive	5	0
False Negative	4	0
Precision	0.97	1.00
Recall	0.98	1.00
F1-score	0.97	1.00

*LN* lymph node

**Fig. 5:**
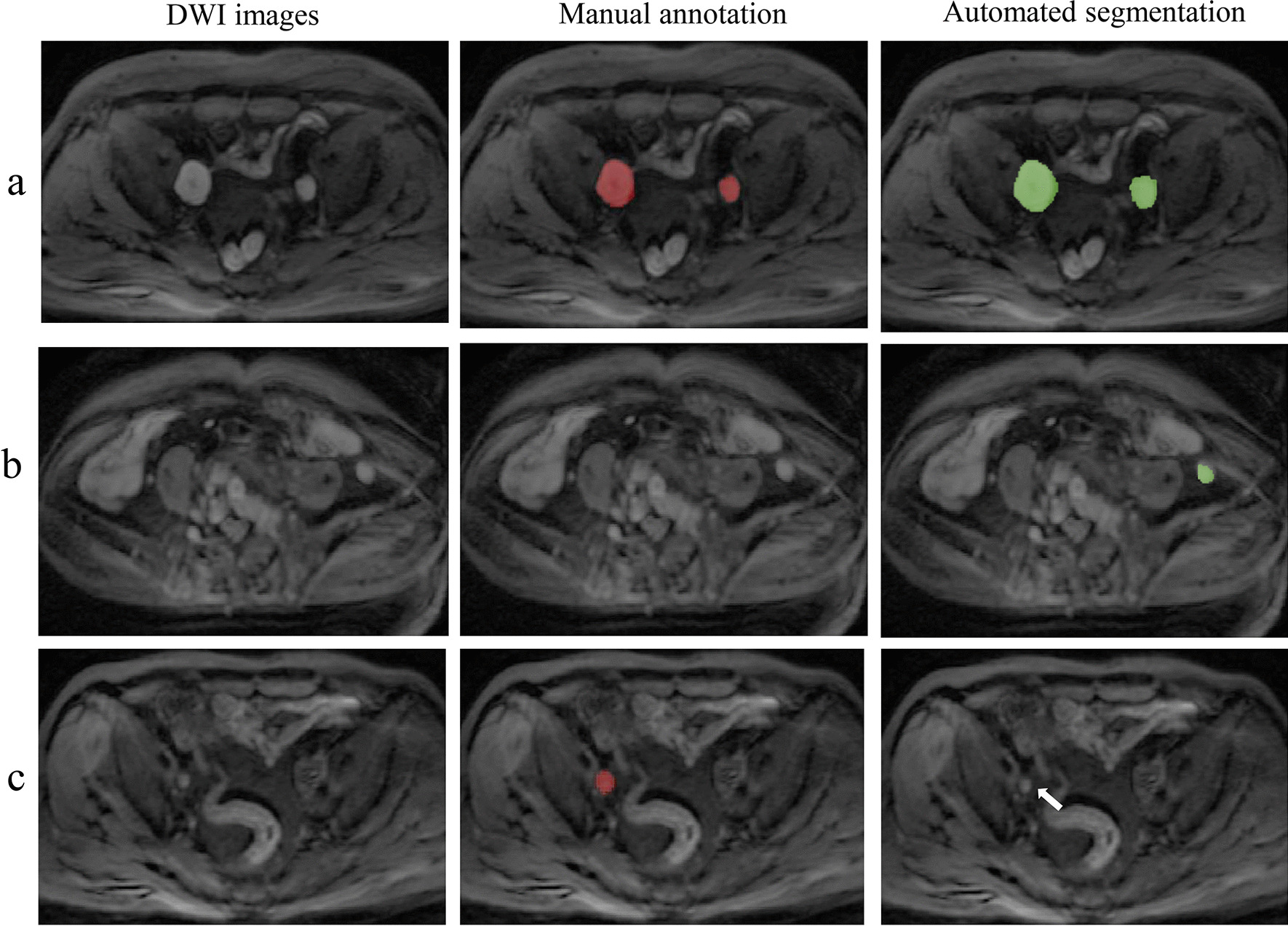
Examples of the detection results of the 3D U-Net for the lymph nodes. The first column represents the DWI images; the second column represents the manual annotations, and the third column represents the automated segmentations. **a** The true positive LNs detected by the model (Green label in automated segmentation); **b** The false positive detection that was a part of the colon (Green label in automated segmentation); **c** The false negative detection that was missed by the model (Arrow points in the automated segmentation)

Figure 5 illustrates examples of the detection results obtained with the developed method for LNs, which shows the TP (Fig. 5a), FP (Fig. 5b), and FN detections (Fig. 5c). Typically, FPs occur due to nonspecific high intensity; FNs usually occur with misattribution of small lesions and insufficient contrast compared with background.

### Segmentation and detection performance in the hold-out test dataset

The hold-out test dataset was collected from a different reference period and there is no cross-contamination between model development dataset and hold-out test dataset. The segmentation and detection accuracy of the model in the hold-out test dataset were assessed at the patient and lesion levels. The sensitivity of the model in identifying PCa patients with or without a suspicious LN was 100% (37/37, 95% CI: 90.5–100%), and the specificity reached 92.5% (37/40, 95% CI: 79.6–98.4%), with an AUC of 0.963 (95% CI: 0.892–0.993).

At the lesion level, the model’s segmentation accuracy for suspicious LNs achieved an average Dice score, TPR, PPV, and VS of 0.83 ± 0.15, 0.80 ± 0.04, 0.81 ± 0.07, and 0.85 ± 0.12, respectively. Based on the segmentation result, the 3D U-Net achieved a detection precision of 0.96, recall of 0.98, and F1-score of 0.97 for suspicious LNs.

Cohen’s kappa coefficients of the LN staging for the model and the resident and expert radiologist were as follows: model *vs.* expert radiologist: 0.922; model *vs.* resident: 0.766; and resident *vs.* expert radiologist: 0.844. The consistency between the model and the expert radiologist was higher than that between the resident and expert radiologist.

## Discussion

In this study, we proposed to develop a 3D U-Net algorithm to achieve automated detection and segmentation of pelvic LNs on DWI images for clinically suspected PCa patients. The results in the testing set and hold-out test dataset confirmed the feasibility of automated LN detection and segmentation, which may aid in LN staging, quantitative measurements of tumour burden and image-guided treatment of PCa patients.

The U-Net algorithm has been widely used for organ and lesion segmentation on MRI images, such as prostate [22, 23] and prostate lesions [24]. However, this method is not easily applied to LN lesions. There is great heterogeneity in the shape and size of lymphadenopathies in the pelvis, making it difficult to discriminate true LN regions from other regions. Compared with that of the background, LN lesions usually account for a small part of the image volume, and this imbalance makes segmentation more difficult. Additionally, the number of FPs that contain nonspecific high-intensity mimics is considerably large, which usually results in lower specificity. Last but not least, the inter- and intraoperator variability of manual annotation remains a longstanding bottleneck for automated image segmentation [25], and there is currently no reliable substitute for manual labelling.

To overcome the imbalanced data problem, several loss functions have been implemented in other studies. For example, the Tversky loss function has been successfully used for the segmentation aneurysmal of the ascending aorta where the anatomy of interest may be very small compared to the background consisting of connective tissue with a wide range of intensity grey values [26]. In this study, we used Dice as the loss function of the 3D U-Net algorithm and manually annotated all visible LNs in the algorithm development dataset, to obtain the specified voxel of the LNs as much as possible. Moreover, considering that LNs are nodular structures for which 3D information is helpful in distinguishing them from tubular structures, because both may show a blob-like structure in 2D images, the 3D U-Net model was selected for segmentation algorithm development [27]. To improve the efficiency and interoperator reliability of data annotation in this study, annotations performed by the two junior radiologists were corrected by an expert radiologist, and the two sets of annotations after modification achieved high Dice scores (Mask 3 *vs.* Mask 4: 0.88 ± 0.06) compared with those before correction (Mask 1 *vs.* Mask 2: 0.75 ± 0.03). The results confirmed the reliability of manual annotation as ground truth for LN segmentation. It is fine to select either Mask 3 or Mask 4 as the ground truth. In this study, we choose Mask 4 as ground truth for the assessment of segmentation for which was performed later than Mask 3.

The algorithm in this study was trained for segmenting all visible LNs on DWI images, whether healthy or metastatic. Given that LNs with a short diameter of more than 0.8 cm were considered to be suspicious for metastasis and of more clinical significance, a cut-off threshold was set to filter out contiguous structures with a short diameter of less than 0.8 cm. These selected LNs were used for the performance assessment of suspicious metastatic LNs. In clinical practice, radiologists usually measure and record the short diameter and volume of the largest LN instead of all metastatic LNs. Therefore, in this study, we also analysed the detection and segmentation performance of the model for the largest LNs. The N-staging was automatically generated based on the quantitative measurements (short diameter and volume) of the largest LN and was sent to the structured report of PCa.

Our results showed that the model achieved good segmentation accuracy of pelvic LNs with an average Dice score and VS of 0.76 ± 0.15 and 0.82 ± 0.14, respectively, in the testing set. Furthermore, the segmentation accuracy of suspicious LNs was significantly higher than that of all LNs (Dice score: 0.85 *vs.* 0.76, *P* = 0.009; VS: 0.82 *vs.* 0.86, *P* = 0.046), which indicated that the segmentation model performed better with large LNs. The average short diameter and volume of the LNs were also measured to quantitively evaluate the segmentation performance. A short diameter is regarded as the reference value to determine the existence of suspicious metastatic LNs, and volume is of high significance for the evaluation of tumour load and response treatment. In our results, both the short diameter and volume of the automated segmentations showed a close correlation with manual annotations on all LNs, suspicious LNs and the largest LNs (all with R > 0.80).

To evaluate the segmentation performance of the model, except for the commonly used overlap-based metrics (Dice, TPR and PPV), the volume-based metric (VS), Spatial distance based metrics (HD, AVD, and MHD) were also defined to assess the segmentation accuracy of the 3D U-Net model. The Dice coefficient, which directly compares the overlap between automated segmentation and manual annotation, is the most commonly used metric for evaluating medical image segmentation [28]. The TPR measures the portion of positive voxels in the manual annotations that are also identified as positive by the automated segmentation, and the PPV indicates the proportion of positive voxels in the manual annotation to the positive voxels in the automated segmentation. VS is a measurement that indicates similarity and considers the volumes of manual segmentation and automated segmentation [21, 29]. With a high VS, the model might be an accurate and convenient tool to assess tumour burden in LNs. The HD and AVD are usually used for contour evaluation. Besides, the AVD is thought to be more stable and less sensitive to outliers than the HD. MHD is a metric for the evaluation of general shape and alignment, which takes into account the correlation of all points in the LNs[21].

The lesion detection approach is proposed based on the segmentation result obtained with the 3D U-Net model. Unlike the segmentation assessment, the detection assessment of LNs was focused on the suspicious LNs in the testing set and hold-out test dataset. This is because the detection of small LNs (short diameter < 0.8 cm) is of little clinical significance. In the hold-out test dataset, in addition to the detection and segmentation assessment of 3D U-Net, we evaluated the clinical application value of the model in the evaluation of suspicious LNs by comparing the consistency of LN staging (N0 or N1) between different readers. Cohen’s kappa coefficient between the model and expert radiologist was significantly higher than that between the resident and expert radiologist (0.922 *vs.* 0.844), which confirmed the feasibility of its clinical application and the possibility of becoming a promising tool for improving the diagnostic accuracy of LN staging for less experienced residents.

Hitesh et al. conducted similar research using U-Net for mediastinal and cervical LNs in CT images. Further, they achieved the differential diagnosis of malignant and benign mediastinal LNs with a fully convolutional network [30] and improved the performance using generative adversarial network and Inception network [31]. Their proposed FCN model has achieved an average sensitivity for the diagnosis of malignant LNs of 90.63% and was then increased to 94.95% by the GAN and Inception network. In our study, we used a fairly large dataset to train a 3D U-Net model and validated the model in a hold-out dataset, the model achieved a high recall (0.98) value in the detection of the suspicious LNs. Our results confirmed the feasibility of the 3D U-Net on automated detection and segmentation of pelvic LNs on DWI images. While some other deep learning approaches (e.g., ENet) [13, 32] may perform better than U-Net. In the future, we would compare the efficiency of these methods regarding the segmentation and detection of LNs.

Serval limitations need to be pointed out in this study. First, the current algorithm cannot provide the exact anatomical location of a particular LN, and a future CNN trained with multiclass annotations of different regional LNs (obturator nodes, external iliac nodes, internal iliac nodes, and common iliac nodes) may be helpful for anatomical location. Second, the 3D U-Net detects, segments and measures LNs larger than 0.8 cm in the short axis but does not render a diagnosis of metastatic LNs. Here, LNs suspicious of metastasis were diagnosed based on their short diameter, which is of reference value to some extent but cannot represent true metastatic LNs. More LN information from PET/CT, MR lymphography or pelvic LN dissection may be necessary for reliable metastatic LN diagnosis. Third, we confined our proof-of-concept study to the pelvic area, but it should be extended to the whole body in the future. A further step towards a faster and comprehensive automated analysis that provides a global tumour burden in PCa patients is the detection and quantification of PCa lesions and skeletal metastases. In addition, although the high Dice score between Mask 3 and Mask 4 indicates that either Mask3 or Mask 4 is acceptable to be the ground truth for the assessment of segmentation, a STAPLE tool may be more convincing to establish the simultaneous ground truth by combining the different segmentations from the clinical experts in a consolidated reference [33]. In the future, we will consider applying the STAPLE tool for ground truth estimation on other segmentation tasks. Last, more data inclusion is necessary for construction of a more robust segmentation model, and multicentre data should be collected to further consolidate the generalization ability of our model. Only patients with PCa were included here, which potentially limits the transferability of our CNN to a broad range of bone metastases of other primary tumors (rectal cancer, bladder cancer, etc.). The application of this method for other types of LNs is needed in the scope of future work.

In conclusion, this study confirmed the feasibility of the 3D U-Net CNN for automated detection and segmentation of LNs on pelvic DWI images. This may present a promising step towards a clinically helpful deep learning-based tool that can provide a comprehensive and objective assessment of tumour burden in PCa patients.

## Data Availability

The datasets used and/or analyzed during the current study are available from the corresponding author on reasonable request.

## Abbreviations

AUC: Area under the curve
CNN: Convolutional neural network
DWI: Diffusion-weighted imaging
FP: False positive
FN: False negative
LNs: Lymph nodes
mpMRI: Multiparametric MRI
PCa: Prostate cancer
PLND: Pelvic lymph node dissection
PPV: Positive predictive value
TN: True negative
TPR: True positive rate
TP: True positive
VS: Volumetric similarity

## Code availability

The code used for the development the algorithm are available from the corresponding author on reasonable request.
